# Supplementary material for: Hedonic pricing analysis for semen of dairy bulls in Brazil
Source: PLoS One. 2022 Apr 19;17(4):e0267109. doi: 10.1371/journal.pone.0267109 (PMC9017886; doi:10.1371/journal.pone.0267109)
Supplement: Attachment — Submitted filename: Response to editor.docx [file pone.0267109.s002.docx]

June 06, 2021

Plos One

Dear Editor:

Please find attached the revised version of our paper (PONE-D-21-07942), entitled “Hedonic pricing analysis for semen of dairy bulls in Brazil”.

We thank you in advance for taking the time to review our manuscript.

To facilitate the review process, each modification we have made is highlighted in the revised version of the manuscript.

Best regards,

Authors.

**Editor review:**

*While I find the topic is interesting, most of the papers cited in the manuscript are too old. I suggest the authors add some recent publications in their reference related to the topic and hedonic price models before I send out the paper for review.*

**Answer:** We agree with the editor’s point. We revised the manuscript to cite papers recently published in the literature on the topic of this paper and hedonic pricing models. The main changes were made in the introduction of the paper between pages 3 and 7 of the revised version of the manuscript. This section has been heavily updated due to the modifications. A modification was also made to section 2 on page 10 of the revised version of the manuscript. The list of references was also updated between pages 21 and 25. We hope that this version is able to proceed in the evaluation process. We are at the editor’s disposal to make additional modifications if necessary.
