# Supplementary material for: Hedonic pricing analysis for semen of dairy bulls in Brazil
Source: PLoS One. 2022 Apr 19;17(4):e0267109. doi: 10.1371/journal.pone.0267109 (PMC9017886; doi:10.1371/journal.pone.0267109)
Supplement: Attachment — Submitted filename: Comments PLOS ONE.docx [file pone.0267109.s003.docx]

This study uses a hedonic price model to analyze the price of semen doses of dairy bulls using the seller’s data from Brazil. The study infers the relationship between the characteristics of the bull and the price of the semen. It also quantifies how the attributes affect the price of semen from milk bulls, and identifies whether proven bulls have higher prices than the others. The authors managed to find there is an additional premium for proven bulls, found that semen doses from Gir bulls proved to be more expensive, and concluded the most valued feature is the dose being sexed. Despite the methodology used in this interesting topic, this article could be further improved in the following aspects.

2.2 model

I felt this part could be more specific in terms of the model specifications. The article would be improved if the authors made it clearer what econometric model finalized used in the manuscript in addition to the economic model and general introduction of the hedonic model. Readers would have to figure it out by reading the results, while the model part should be discussing it more clearly.

4. Conclusions

The ending of the manuscript is a bit too quick. As the ending part, the manuscript would benefit more from an extended discussion of how the current results compared with the previous ones, what is the significant contribution of the current study, and why it is so important. Those three components could become a Discussion & Implication section, in addition to the current final summary of the general results. Right now, it lacks the discussion of the broader impacts of the study, and readers may feel unsure why we should even care about this study.
