# Supplementary material for: Hedonic pricing analysis for semen of dairy bulls in Brazil
Source: PLoS One. 2022 Apr 19;17(4):e0267109. doi: 10.1371/journal.pone.0267109 (PMC9017886; doi:10.1371/journal.pone.0267109)
Supplement: Attachment — Submitted filename: Response to Reviewers.docx [file pone.0267109.s004.docx]

February 03, 2022

Zhifeng Gao

Academic Editor

Plos One

Dear Editor:

Please find attached the revised version of our paper (PONE-D-21-07942R1), entitled “Hedonic pricing analysis for semen of dairy bulls in Brazil.” With this submission, we are also enclosing our replies to all the reviewer comments.

We thank you for taking the time to review our manuscript, and we hope that this version is now acceptable for publication in *Plos One*. All reviewer comments were considered. The manuscript was also proofread. We look forward to your final decision.

To facilitate the review process, each modification we have made is highlighted in the new file.

Please do not hesitate to contact me if any additional information is needed at this stage of the process.

Best regards,

Authors.

*Dear Reviewer,*

*Thank you for your comments and suggestions. We would like to emphasize that all comments have been considered and that we are confident that all requests for modifications have been met. You will find a specific answer in red to each of the comments below. In addition, each modification is highlighted in the revised version of the manuscript.*

*Regards,*

*Authors.*

**Reviewer #1:**

This study uses a hedonic price model to analyze the price of semen doses of dairy bulls using the seller’s data from Brazil. The study infers the relationship between the characteristics of the bull and the price of the semen. It also quantifies how the attributes affect the price of semen from milk bulls, and identifies whether proven bulls have higher prices than the others. The authors managed to find there is an additional premium for proven bulls, found that semen doses from Gir bulls proved to be more expensive, and concluded the most valued feature is the dose being sexed. Despite the methodology used in this interesting topic, this article could be further improved in the following aspects.

*Answer: Thank you for your comment. All points indicated in the review were considered in the revised paper.*

2.2 model

I felt this part could be more specific in terms of the model specifications. The article would be improved if the authors made it clearer what econometric model finalized used in the manuscript in addition to the economic model and general introduction of the hedonic model. Readers would have to figure it out by reading the results, while the model part should be discussing it more clearly.

*Answer: Thank you for your comment. We made substantial changes to the presentation of the methodology to reflect your suggestion. Section 2.2 has been completely rewritten to explain the model and the estimated equations. The revisions appear on pages 9 to 18 of the new version of the manuscript, and are as follows:*

The method for assessing the value of product characteristics or components has changed since Waugh’s (1928) work, in which the objective was to evaluate how the quality factors (e.g., color, size, and condition) of some products sold at fairs (e.g., tomatoes, asparagus, and cucumbers) were correlated with their prices. The analysis indicated high correlation coefficients (in module), demonstrating that these factors were associated with product prices. This seminal effort opened fronts for developments based on more sophisticated statistical techniques, with application to various themes, such as milk (Hiller et al., 1980), cotton (Bowman and Ethridge, 1992), fish (McConnell and Strand, 2000), and wine (Oczkowski, 2001). Additionally, economic theory provided the basic foundation for guiding agents’ behavior in market situations. Therefore, the analysis that is intended to be developed in this paper is methodologically justified by the analytical capacity of the hedonic price approach, with applications to evaluate various markets, and its development in the light of the economic theory that guides it. The main theoretical aspects are described below. It is interesting to note that Waugh’s (1928) analysis was based on information collected via field research that focused on the commercialization segment. This research was conceived in the same context.

The theoretical background underlying the analysis is the economic theory of hedonic prices, comprising representative consumer decisions that consider the implicit characteristics of products in the utility maximization process. Any differentiated product can be considered a set of several quality attributes that consumers independently assess at the time of purchase. Thus, given a representative consumer’s budget constraints, they consider the attributes of the product during the consumption decision-making process and obtain utility from that (Lenz et al., 1991). For instance, dairy producers, when purchasing bovine semen, expect to maximize utility by considering the product characteristics, such as tested semen and sexed semen. Furthermore, semen buyers may experience different satisfaction levels if they are aware of the different genetic transmission capabilities of the semen, such as the potential for higher milk volume production or higher protein content.

Lenz et al. (1991; 1994) formalized a model based on consumer theory to evaluate the behavior and milk consumption decisions of a representative agent. The idea presented in these works is identical to that in this study, albeit the present research applies the idea to the bovine semen market. Works prior to Lenz et al. (1991; 1994) formalized a model based on the theory of the firm, since analyses of the milk market focused on production techniques and the use of inputs to define the profit maximization behavior of a representative agent (e.g., Kirkland and Mittelhammer, 1986; Gillmeister et al., 1996; Buccola and Iizuka, 1997). Examples of this line of analysis are the employment of hedonic price models to assess the implicit effect of the use of each production input (e.g., Rudstrom; 2004) or the use of milk as a production input to produce another product, such as cheese or yogurt (e.g., Hillers et al., 1980).

Therefore, the hedonic pricing model aims to establish a relationship between the characteristics of a product and its price, that is, how much more or less the consumer (in this case, milk producers) is willing to pay for a particular attribute. Although these prices are not explicitly expressed in the market, they can be estimated using a regression model capable of directly expressing the price of a product as a function of directly or indirectly observable quality characteristics (Carlucci, 2013). At the end of the formalization presented below, the equations to be econometrically estimated will be derived.

Based on the formulations of Lancaster (1966) and Rosen (1974) as well as the derivations of Lenz et al. (1991; 1994), Rudstrom (2004), and Ballco and de-Magistris (2018), the hedonic price function considers a set of attributes that can be represented by a *Z* vector of characteristics:

$Z=\left( Z_{1},Z_{2},\ldots,Z_{n} \right)$	(1)

Thus, the utility function is specified according to the characteristics of the product instead of the quantity of the product consumed. A representative agent’s utility function, considering that *z_j_* is the attribute *j* of the consumed product, is expressed as:

$U=U\left( z_{1},z_{2},\ldots,z_{n},\alpha\right)$	(2)

where *α* is a fixed parameter of consumer preferences. It is assumed that the magnitude of a characteristic is a function of the product quantity and the number of product characteristics:

$z_{j}=F\left( v_{1},v_{2},\ldots,v_{i},z_{j1},z_{j2},\ldots,z_{jn} \right)$	(3)

where *v_i_* is the quantity of product *i* and *z_ij_* is the magnitude of characteristic *j* in a unit of product *i*.

Therefore, the problem of maximizing consumer utility is represented by the Lagrangian maximization problem:

$L=U\left( z_{1},z_{2},\ldots,z_{n},\alpha\right)-\lambda\left( \sum_{i=1}^{n} p_{i}v_{i}-I \right)$	(4)

where *p_i_* is the price of the product *i*, *I* is the representative agent’s income, and *λ* is the Lagrange multiplier. The first order conditions in Equation (4) yield the following result:

$\frac{\partial L}{\partial v_{i}}=\sum_{j} \frac{\partial U}{\partial z_{j}}\frac{\partial z_{j}}{\partial v_{i}}-\lambda p_{i}=0$	(5)

Equation (5) provides the necessary elements for analyzing the value of the product characteristics. The term $\frac{1}{\lambda}\frac{\partial U}{\partial z_{j}}$ represents the implied price or marginal value of characteristic *j*. The term $\frac{\partial z_{j}}{\partial v_{i}}$ is the marginal value that characteristic *j* gives a unit of product *i*. Isolating *p_i_* in Equation (5), we obtain the result Rosen (1974) proposed, which specifies prices as a function obtained at the point of equilibrium between demand and supply of product *i*:

$p_{i}=G\left( Z_{j} \right)$	(6)

where $G\left( . \right)$ is a function of hedonic prices specified in general terms of the attributes associated with product *i*.

Equation (6) indicates that the price *p* paid is a function of the marginal monetary value of the *j*-th attribute *Z*. The implied marginal price that a consumer is willing to pay for attribute *j* is equal to the marginal cost the producer incurs to offer it (Costanigro and McCluskey, 2011).

Lenz et al. (1991) presented a concept capturing consumer perception of the value of the product as reflected in the price paid to acquire it. For example, if a consumer perceives *n* characteristics of a product, then the amount paid for the product can be broken down as follows:

$p=\sum_{j=1}^{n} V_{j}Q_{j}$	(7)

where *V_j_* is the implicit value or price the consumer assigns to a unit of characteristic *j*, and *Q_j_* is the number of units of characteristic *j*.

An empirical representation of the decomposition of the product price is conceptualized in Equation (7), with a view to statistical estimation. Due to the data structure, three models are estimated. The main reason for estimating three different models is the limited availability of information about the progeny test for the Guzerá breed. The estimated models are described in Table 2.

**Table 2. Models and variables used in the estimations**

| **Models** | **Estimations** | **Dependent variable** | **Independent variables** |
| --- | --- | --- | --- |
| Model 1 | Three estimations: 1) one for Gir, 2) one for Girolando, and 3) one considering Gir + Girolando | *Price* | *Proven* |
| Model 2 | One estimation | *Price* | *Breeds* (Girolando and Guzerá), *AI firms* (CRV, ABS, SEMEX) (Note: Gir from ALTA was considered the baseline product.) |
| Model 3 | Two estimations: 1) one for Gir and 2) one for Girolando | *Price* | *Number of Herds, PTA - Milk (volume), PTA - Age at First Birth, PTA - Fat (kg), PTA - Protein (kg), Sexed, Alive* |

Source: Own elaboration based on research data.

The first model to be estimated aimed only to identify the implicit premium of the semen price with respect to the progeny test. Three estimations were performed considering the specification of the first model: one for the Gir breed, one for the Girolando breed, and one considering the bulls of both breeds. Following the nomenclature Bimbo et al. (2016) used, there is one variable to be considered in Equation (7), sub-vector *Z^T^*, which indicates whether the bulls of each breed are proven. Due to the decrease in informational asymmetry and proof of the bull’s genetic transmission capacity, it is expected that the semen dose from proven animals will have a higher price.

The second model aimed to explain the existence of a price differential for breeds and artificial insemination (AI) firms. Therefore, the specification of Equation (7) has two attributes: sub-vector *Z^B^* for breeds and sub-vector *Z^A^* for AI firms. According to Ladd and Suvannunt (1976), a product can be described according to its inherent attributes, thereby generating the need for a basic unit of analysis. For this analysis, the Gir breed and the AI firm ALTA were chosen as the baseline characteristics of the semen. Alternatively, the complete vector of characteristics could be considered in the estimation, excluding the constant. Information about this breed and the selected AI firm form the characteristic product (semen), and the difference in price obtained is relative to the price of semen from the Gir bull negotiated by the AI firm ALTA. This procedure also aims to avoid multicollinearity.

The third model was developed to identify the specificities of each breed. The attributes evaluated in this model were computed based on the progeny test. Considering the specification of the third model, one was estimated for each breed. As the test data for the Guzerá breed were not obtained, in the specification of Equation (7) for the Gir and Girolando breeds, the following characteristics were considered: *Z^R^*, *Z^P^*, *Z^S^*, and *Z^L^*. The sub-vector *Z^R^* represents the number of herds. It is expected that the greater the number of herds, the higher the price of the semen. The sub-vector *Z^P^* represents the PTAs: PTA – Milk (volume), PTA – Age at First Birth, PTA – Fat, and PTA – Protein. Given that the payment model adopted by dairy companies in the country favors production volume, it is expected that, across all breeds, the doses from the bulls with the best results for PTA – Milk (volume) will be more expensive. The sub-vector *Z^S^* indicates whether the semen is sexed or not. Sexed semen is expected to have a positive effect on semen price, since this attribute guarantees female offspring. Finally, the sub-vector *Z^L^* indicates whether the semen came from a bull that was alive. The effect of this attribute on semen price is ambiguous. If the market understands that the semen was from a bull that is not alive, this represents a shortage, and the effect on the price of semen tends to be positive; if the bull is alive, then the effect is negative. If the market understands that the semen from a (not) live bull is of better quality than semen from bulls from past generations, the effect of the bull not being alive (or being alive) on the price of the semen tends to be negative (positive).

The relationship between the price of the product and its attributes as established in Equation (6) has no theoretically defined functional form. Thus, the literature suggests the adoption of linear and semi-logarithmic functions and the Box-Cox transformation (Costanigro and McCluskey, 2011). As an initial step, the literature suggests performing the Box-Cox test as a parameter for indicating the best functional form. The Box-Cox test performed in the present study did not indicate the functional form with more robust results. Thus, we followed the recommendation to use the semi-logarithmic specification of the hedonic price equation (Rudstrom, 2004; Costanigro and McCluskey, 2011; Loke et al., 2015; Bimbo et al., 2016; Yang and Dharmasena, 2020; Qi and Ploeger, 2021; Jiang et al., 2021).

To estimate Models 1, 2, and 3, we have the following specifications, respectively:

$ln\left( p_{i} \right)=\beta_{0}+\beta_{t}Z^{T}+\varepsilon_{i}$	(8)

$ln\left( p_{i} \right)=\beta_{0}+\sum_{b=1}^{B} \beta_{b}Z_{b}^{B}+\sum_{a=1}^{A} \beta_{a}Z_{a}^{A}+\varepsilon_{i}$	(9)

$ln\left( p_{i} \right)=\beta_{0}+\beta_{r}Z^{R}+\sum_{p=1}^{P} \beta_{p}Z_{p}^{P}+\beta_{s}Z^{S}+\beta_{l}Z^{L}+\varepsilon_{i}$	(10)

where *β* represents the coefficients to be estimated, which are the implicit values associated with the different attributes, and *ε_i_* is the error term. In Equation (8) (Model 1), the sub-vector *Z^T^* indicates whether the progeny test was performed. In Equation (9) (Model 2), *Z^B^* is the breeds’ sub-vector of attributes indexed by *b*, where *b = 1, ..., B* (or *Z^B^ = Girolando, Gir, Guzerá*), and *Z^A^* is the AI firm’s sub-vector of attributes indexed by *a*, where *a = 1, ..., A* (or *Z^A^ = ALTA, CRV, ABS, SEMEX*). In Equation (10) (Model 3), the sub-vector *Z^R^* is the attribute of the number of herds, *Z^P^* is the sub-vector of attributes of PTAs indexed by *p*, where *a = 1, ..., P* (or *Z^P^ = PTA – Milk (volume), PTA – Age at First Birth, PTA – Fat, PTA – Protein*), *Z^S^* is the sub-vector of attributes that indicates whether the semen is sexed, and *Z^L^* is the sub-vector that indicates whether the semen is from a live bull.

The marginal value of each attribute is its implicit price, and it represents a change in the price given a marginal change in the characteristic (Rudstrom, 2004). Considering that the variable is continuous, the marginal value of this characteristic is the partial derivative of Equations (8), (9), and (10) in relation to that characteristic:

$\frac{\partial\ln\left( p \right)}{\partial z_{i}}=\left( \frac{\partial p}{\partial z_{i}} \right)\left( \frac{1}{p} \right)=\beta_{i}$	(11)

In addition to calculating the implicit marginal price (Equation (11)), the marginal effect of each attribute on prices in percentage terms is calculated. Models that aim to explain the percentage impact on the dependent variable by means of dummy variables need to be corrected, as the variable is not directly related to a percentage. To accomplish this, the correction that Kennedy (1981) proposed, which is suitable for the semi-logarithmic form of adjustment of the variables in terms of elasticity, was used, as follows:

$\hat{p}=100\times\left\{ \left[ exp\left( \hat{c}-\frac{1}{2}V\left( \hat{c} \right) \right) \right]-1 \right\}$	(12)

where $\hat{p}$ is the marginal price of the attribute, $\hat{c}$ is the estimated coefficient, and $V\left( \hat{c} \right)$ is the variance of $\hat{c}$.

Additionally, obtaining the values of the premiums or relative discounts of the attributes facilitates a comparison of the results of this study with those of other studies that may have been developed for other analysis contexts, such as different markets or other countries, breeds, or periods. More recent studies on the milk market have prioritized the relative effects of premiums on product attributes (Loke et al., 2015; Bimbo et al., 2016; Ballco and de-Magistris, 2018).

For the estimation of Equations (8), (9), and (10), we relied on ordinary least squares (OLS), considering all the characteristics described in Table 2. To identify the existence of heteroscedasticity in the estimated equations, the Breusch-Pagan/Cook-Weisberg test was performed. In cases where heteroscedasticity was found, White’s robust correction process was applied.

4. Conclusions

The ending of the manuscript is a bit too quick. As the ending part, the manuscript would benefit more from an extended discussion of how the current results compared with the previous ones, what is the significant contribution of the current study, and why it is so important. Those three components could become a Discussion & Implication section, in addition to the current final summary of the general results. Right now, it lacks the discussion of the broader impacts of the study, and readers may feel unsure why we should even care about this study.

*Answer: Thank you for your comment. We agree with your suggestion. To implement the recommendation, a section was inserted before Conclusions to reinforce the (non)existence of previous results in the literature and clarify the study’s importance and contributions. It is worth mentioning that this section may seem repetitive, as the elements the reviewer requested also appear elsewhere in the paper. The text of the new section, Discussion and Implications, is given below. It can be found on pages 25 to 27 of the revised paper:*

**4 Discussion and Implications**

**Techniques based on selection and genetic improvement are increasingly being used in the production process, especially in the livestock environment in Brazil. Genetic herd mapping is among the most commonly used techniques to increase milk production and productivity. Mapping makes it possible to identify the main genetic characteristics that can positively influence milk production. It is not only done with cows but also with semen-supplying bulls that can genetically transmit traits to female offspring, affecting their potential to produce milk. Large volumes of data are being collected on the possible attributes of bulls and their semen, such as the transmission of the ability to produce greater volumes of milk and sexed semen. However, the market still prices bulls’ semen without reference to the intrinsic value of each attribute linked to the semen. This paper contributes to this aspect by providing information about the implicit prices attributed to the characteristics of bulls and their semen. AI is being increasingly used by dairy producers seeking to increase production and obtain optimum production levels to remain competitive in the market; bovine semen is one of the main inputs in AI.**

**This paper provides essential information, especially for dairy producers that buy bovine semen, about the value of semen attributes. Specifically, the information presented here can help them plan how much semen to buy from which bulls and how much to pay for sexed semen that has the greatest potential to increase milk volume or milk solids content. One of this study’s main findings is that only the testing of the bull has a positive effect on the price of semen. This was expected because testing is an important step in genetic improvement and contributes to reducing the asymmetry of information about bulls’ characteristics. There is no similar finding in the literature. Other evidence emerging from this research is that semen prices can vary between breeds and AI firms. Each breed has a different vocation for milk production, and this can impact the price of semen. Moreover, milk producers who want to buy semen should be aware of the various companies that sell and perform insemination, as there may be significant price differences between them. This is also new evidence in the literature.**

**It is also worth noting that for the breeds analyzed in Brazil, sexed semen tended to have a higher price, given the ability to transmit the potential to produce higher volumes of milk. This result is similar to that obtained by Richards and Jeffrey (1996), but it is worth remembering that this study was conducted in another country and involved a different breed. Nevertheless, the evidence presented in this paper indicates that there is a greater and more significant appreciation in the Brazilian market for the ability to generate higher volumes of milk compared to higher milk solids content. This is new evidence in the literature on Brazilian dairy bull semen prices that can inform dairy producers’ semen purchase decision making. It is worth stressing, again, that given intensifying market competitiveness, genetic improvement techniques are essential for producers. According to an ASBIA (2021) report, in the second half of 2021, 62% of Brazilian municipalities used doses of semen from dairy bulls, and the number of doses sold grew by more than 30% in 2020. Therefore, all the evidence presented in this paper contributes to decision making in a multimillion-dollar market concerning an essential input for the milk production process in Brazil.**

*As the conclusions of the paper are mainly suggestions for dairy farmers, in light of the findings, the following text has been added at the beginning of the conclusions section, on page 27 of the revised paper:*

The conclusions of this paper suggest means by which the bovine semen market can incentivize milk producers, based on the study’s findings.

*Dear Reviewer,*

*Thank you for your comments and suggestions. We would like to emphasize that all comments have been considered and that we are confident that all requests for modifications have been met. You will find a specific answer in red to each of the comments below. In addition, each modification is highlighted in the revised version of the manuscript.*

*Regards,*

*Authors.*

**Reviewer #2:**

Overall Paper Comments:

The authors use a hedonic pricing model to determine what value buyers of dairy bull semen place on certain characteristics of the semen given its genetic merit for milk production and quality. It was determined that milk volume is the pace setter for determining semen value. The research idea is not novel in and of itself. However, it does likely contribute to the literature from a regional aspect in that this information could be important for Brazilian dairy producers, sellers of semen, and milk purchasers. The findings of this research were not unexpected, but it provides the foundation on which further research can be based. Similarly, this foundation could lead to changes within the Brazilian dairy industry if milk components become more desirable by milk purchases than just volume of fluid milk. The reviewer realizes the authors were instructed to expand on the introduction and add some literature review better form the research objective. However, the introduction was a little long for my taste. The editor and the authors can discuss that topic if it is found to be worthy of discussion. Otherwise, the paper is acceptable. A few specific errors have been noted, and there may have been some overlooked during the review.

*Answer: Thank you for your comment. You are correct. The editor requested the expansion of the literature review.*

Specific Comments:

Page 3, Line 4

Should be “head” instead of “heads”.

Page 4, Line 7

Should be “possible to obtain instead of “possible obtain”.

Page 4, Line 21

Delete “that” following the word “but”.

Page 5, Line 14

A comma is needed prior to “because”.

Page 6, Line 15

Should be “the Holstein breed” instead of “Holstein breed”.

*Answer: Thank you for your careful reading. All errors indicated have been corrected in the revised paper.*

*Additional comment:*

*The revised paper was modified to provide more detail on the estimated econometric models. These are given in Section 2.2., from pages 9 to 18. In addition, a discussions and implications section was also added between Results and Conclusions. This section, on pages 25 to 27, provides additional information about the contributions and implications of the study.*
