# Supplementary material for: Hedonic pricing analysis for semen of dairy bulls in Brazil
Source: PLoS One. 2022 Apr 19;17(4):e0267109. doi: 10.1371/journal.pone.0267109 (PMC9017886; doi:10.1371/journal.pone.0267109)
Supplement: Attachment — Submitted filename: Comments Plos one second round 0331.docx [file pone.0267109.s005.docx]

The authors addressed my last round of concerns by improving the model specification and conclusion by adding many materials and discussions. The revised manuscript is in a good shape, and I would suggest accepting it for publication.
